# Behaviors Related to Limiting Fat Intake among Young Adults in Saudi Arabia

**DOI:** 10.3390/nu15214540

**Published:** 2023-10-26

**Authors:** Walaa Abdullah Mumena, Lamar Haitham Owaidhah, Ruba Abdulrahman Alsaadi, Nada Mohammed Aljuhani, Laila Sulaiman Almehmadi, Hebah Alawi Kutbi

**Affiliations:** 1 Clinical Nutrition Department, College of Applied Medical Sciences, Taibah University, P.O. Box 344, Madinah 42353, Saudi Arabia; 2 Clinical Nutrition Department, Faculty of Applied Medical Sciences, King Abdulaziz University, P.O. Box 80215, Jeddah 21589, Saudi Arabia; hkutbi@kau.edu.sa

**Keywords:** behaviors, limiting fat intake, fat intake, young adults, Saudi Arabia

## Abstract

Young adults tend to frequently consume foods that are high in fat. Efforts to limit the consumption of fat among the Saudi population have been initiated; however, data concerning current behaviors related to limiting fat intake are lacking. We aimed to explore behaviors related to limiting fat intake and to investigate the association with fat intake among young adults in Saudi Arabia. In this cross-sectional study, a total of 305 students aged ≥19 years were recruited from Taibah University, Madinah. Face-to-face interviews were conducted to collect data concerning sociodemographic status, behaviors related to limiting fat intake, and fat intake. The median score of behaviors related to limiting fat intake was significantly higher among females and supplement users compared to other groups. Healthy weight, overweight, and obese students reported a significantly higher score of behaviors related to limiting fat intake, compared to underweight students. Males who reported making an effort to limit the consumption of fatty foods consumed less total fat, saturated fat, monounsaturated fat, and polyunsaturated fat, while those who reported reading the nutrition fact labels of food products consumed more polyunsaturated fat. Females who reported reading nutrition fact labels consumed less saturated fat. Efforts to limit fat intake have been noted especially among females; however, these efforts were not linked to fat intake among young adults in Saudi Arabia.

## 1. Introduction

Fat is a valuable macronutrient that contributes greatly to energy, providing 9 kilocalories per gram, and is the most efficient store of energy in the body [1]. Fat has many important physiological functions, including enhancing the palatability of foods and promoting the absorption of fat-soluble vitamins [2,3]. However, the World Health Organization (WHO) recommends that dietary fat intake should not exceed 30% of the total energy consumed [4]. There are several types of fat found in food, including trans fatty acids, saturated fatty acids (SFAs), and unsaturated fatty acids (UFAs).

Certain types of fat have been found to be associated with health benefits, whereas the overconsumption of others can negatively impact health. UFAs are predominantly found in oils derived from plants and fish and can be either monounsaturated (MUFA) or polyunsaturated (PUFA). The consumption of these fats in moderation is associated with numerous health benefits, including improving cardiovascular health, lowering blood cholesterol levels, and aiding in weight loss [5,6]. SFAs are found in animal foods, including meat and dairy products, as well as some plant sources, such as palm and coconut oils. Trans fatty acids are generally found in ultra-processed food, fast food, and fried food. Recent evidence revealed that limiting the intake of SFAs, combined with an increased intake of PUFAs, can lower the risk of cardiovascular disease [7]. Additionally, no association has been found between fat intake and an increased risk of stroke, colorectal, and ovarian cancers [8,9,10], yet excessive fat intake has been found to be linked to an increased risk of breast cancer [11].

The Saudi Food and Drug Authority has established food labeling-related policies as an effort to limit the consumption of fat, to prevent adverse health effects at the national level [12]. However, data regarding fat intake and its influential factors among the Saudi population are still limited. It is necessary to monitor fat intake in the Saudi population in order to design effective interventions that aim to limit the intake of fat. This study was designed to explore behaviors related to limiting fat intake and investigate the association with fat intake among young adults in Saudi Arabia.

## 2. Materials and Methods

### 2.1. Study Design and Population

In this cross-sectional study, healthy young adults (≥19 years old), enrolled in an undergraduate program at Taibah University, Madinah, Saudi Arabia, were recruited conveniently. The exclusion criteria were food allergy, chronic diseases, following a dietary regimen, incomplete dietary data, being pregnant, and the use of any medications that could affect weight status. Based on the equation proposed by Charan and Biswas for cross-sectional studies, we needed to recruit a minimum of 138 male students and 138 female students: sample size = Z1-/2 (SD) 2/d2, where Z1-/2 = 1.96 (95% CI), expected SD = 30 g of fat, and d = 5 g of fat [13].

The approval to conduct this study was obtained from the ethical review board of the College of Applied Medical Sciences, Taibah University (certificate no. 2023/151/202 CLN). Informed consent was obtained from all subjects involved in the study.

### 2.2. Data Collection

Data were collected between December 2022 and January 2023. A total of 320 questionnaires were distributed to students (160 copies for the male section and 160 copies for the female section). Multiple stations were assembled in different buildings in main campuses (both male and female). Banners, that included an invitation to be part of the study, a summary of the research aim, and a barcode to visit the link that allowed the students to read more information about the study, were available in each station. Each questionnaire included a section concerning sociodemographic data (age, sex, family monthly income, and year of study), lifestyle-related practices (sleeping hours per day (<7 h/d, ≥7 h/d [14]; daily media use (≤2 h/d, >2 h/d); supplement use (no, yes); height and weight), and a section that focused on assessing fat intake and behaviors related to limiting fat intake. The data collection team received multiple training sessions to ensure the accuracy of the data collected.

### 2.3. Assessment of Anthropometrics

Height and weight were self-reported by students both in male and female sections. The anthropometric data reported were used to calculate a BMI for each student. The WHO cutoff points were used to define the weight status as follows: underweight (BMI < 18.5 kg/m^2^); healthy weight (BMI 18.5–24.9 kg/m^2^); overweight (BMI 25–29.9 kg/m^2^); and obese (BMI ≥ 30 kg/m^2^) [15].

### 2.4. Assessment of Fat Intake

Data concerning fat intake were assessed using an adapted version of a food frequency questionnaire (FFQ) that was developed by Rohrmann and Klein in 2003 [16]. The FFQ aims to assess fat intake in grams within the past 30 days. The FFQ was modified based on food items commonly consumed by Saudis; modifications were made and validated by two experts in the field of nutritional assessment. The FFQ included five food groups and 23 food items: (1) beef/lamb/camel; (2) chicken (not fried); (3) fried food (fried chicken, fried fish, kubba, samosa, fried vegetables, and French fries); (4) egg (boiled, fried, or scrambled); (5) fish (salmon or sardine); (6) processed meat (salami, hotdog, or cold cuts); (7) sausages; (8) cakes/pastries; (9) chocolate; (10) cookies/biscuits; (11) cream; (12) full-fat cheese/cream cheese; (13) full-fat milk; (14) ice-cream; (15) nuts; (16) tahini/Halawa tahinia; (17) mayonnaise; (18) salad dressing; (19) ghee; (20) butter; (21) plant fats (olive oil, canola oil, corn oil); (22) avocado; and (23) chips. The frequency of consumption provided in the FFQ was as follows: daily (once a day, two to three times per day, four to five times per day, six or more times per day); weekly (once per week, two to four per week, five to six per week); monthly (one to three per month, less than once per month). Answers were recorded in a card copy of the FFQ, then responses were entered into an Excel sheet, to calculate the intake of fat per gram per day.

### 2.5. Assessment of Behaviors Related to Limiting the Intake of Fat

Behaviors related to limiting fat intake were assessed using a 5-item questionnaire. Students were asked if any of the following behaviors were performed as an effort to limit fat intake: (1) read nutrition fact labels; (2) consume low-fat milk and dairy products; (3) reduce the use of fats and oils while cooking; (4) limit the consumption of fast food; (5) limit the consumption of fatty foods (e.g., fried food items and fatty meats). Responses to each of the five items were “no”, which was coded as zero, and “yes”, which was coded as one.

### 2.6. Statistical Analysis

The Statistical Package for Social Sciences was used for data analyses (IBM SPSS Statistics for Windows, Version 20.0, IBM Corp., Armonk, NY, USA). Continuous variables are presented as mean ± standard deviation and median (interquartile range), whereas categorical data are presented as frequency (percentage). The Shapiro–Wilk test was used to evaluate the normality of the distribution of continuous variables (total fat, SFA, MUFA, and PUFA), which indicated skewed data for all variables. The median intake of all types of fat was compared among male and female students using the Mann–Whitney test. Mann–Whitney and Kruskal–Wallis tests were used to compare the median scores of behaviors related to limiting fat intake across the different groups. Dunn’s post hoc test was used to further explore the significant findings obtained using Kruskal–Wallis test. Fisher’s exact test was used to assess the association between categorical variables. A simple linear regression analysis was performed to explore the association between behaviors related to limiting fat intake (the independent variable) and fat intake, including total fat, SFAs, MUFAs, and PUFAs (the dependent variable). Additional linear regression models were tested to explore the link between the total score of behaviors related to limiting fat intake (independent variable) and fat intake (dependent variable). The confidence level used to assess the significance of the test performed was set at 95%.

## 3. Results

### 3.1. Sample Characteristics

The total sample included in this study consisted of 305 students (152 male students and 153 female students), after excluding 4.69% (*n* = 15), wherein 4.38% (*n* = 14) were excluded due to incomplete data and 0.31% (*n* = 1) due to being on diet restrictions. Approximately half of the students were aged between 21 and 23 years (47.9%, *n* = 146), and most of the students reported being single (95.7%, *n* =292). Thirty-one percent of the students (*n* = 95) reported a monthly family income of less than 6000 Saudi Riyal (SR) and 93.1% of the students (*n* = 284) were unemployed. Ninety-five percent of the students (*n* = 290) reported living with their families. Over half of the students (58.7%, *n* = 179) reported being in their third and fourth year of the program, and 54.4% (*n* = 166) of the students were enrolled in a non-health-related program. Sixty-three percent of the students (*n* = 193) reported sleeping for 7 h or more per day. Only 5.60% (*n* = 17) reported using media within the recommendation of ≤2 h per day. A few students (20.0%, *n* = 61) reported using dietary supplements. Nineteen percent of the students (*n* = 57) were underweight. Over half of the students were within the healthy weight range, whereas 29.8% of the students (*n* = 91) were overweight or obese (Table 1). The median intake of total fat, SFA, MUFA, and PUFA was 84.8 g/d (56.7–128), 30.9 g/d (20.2–47.1), 28.4 g/d (17.9–44.1), and 15.1 g/d (9.13–23.9), respectively. Detailed data concerning fat intake among male and female students are provided in Appendix A.

### 3.2. Behaviors Related to Limiting Fat Intake

Twenty-two percent of the students (*n* = 67) reported reading the nutrition fact labels on food products as an effort to limit their fat intake, while 13.8% of the students (*n* = 42) reported consuming low-fat milk and dairy products. Approximately one-third of the students (*n* = 99) stated they reduced their use of fat and oils while cooking, whereas 48.2% (*n* = 147) and 37.4% (*n* = 114) reported limiting their consumption of fast food and fatty food (fried food items and fatty meat), respectively. A significantly higher proportion of females reported reducing their use of fats and oils while cooking as well as limiting their consumption of fatty foods (e.g., fried food items and fatty meats) compared to male students (62.6% vs. 37.4% and 66.7% vs. 33.3%, respectively, *p* < 0.001 for both). The mean total score of behaviors related to limiting fat intake was 1.66 ± 2.08. Detailed descriptive data concerning behaviors related to limiting fat intake are provided in Table 2.

### 3.3. Association between Characteristics of the Study Sample and Behaviors Related to Limiting Fat Intake

The median total score of behaviors related to limiting fat intake was significantly higher among females compared to male students (2.00 (1.00–3.00) vs. 1.00 (0.00–2.00), *p* < 0.001). In addition, the median total score of behaviors related to limiting fat intake was significantly higher among students who used supplements compared to students who did not use supplements (2.00 (1.00–3.00) vs. 1.00 (0.00–2.00), *p* = 0.002). There was a significant association between weight status and behaviors related to limiting fat intake (*p* = 0.003). The results of the post hoc test revealed that the total score of behaviors related to limiting fat intake was significantly higher among students whose weight status was within the healthy weight range, overweight, or obese, compared to underweight students (*p* = 0.001, *p* = 0.002, and *p* = 0.010, respectively). The total score of behaviors related to limiting fat intake was similar across all other groups (see Table 3).

### 3.4. Association between Fat Intake and Behaviors Related to Limiting Fat Intake

A simple linear regression analysis indicated that limiting the consumption of fatty food (e.g., fried food items and fatty meats), among males, was associated with a lower intake of total fat (beta (B) = −68.9, standard error (SE) = 27.9 [95% confidence interval (CI): −124 to −13.6]), SFA (B = −23.3, SE = 9.26 [95% CI: −41.6 to −5.02]), MUFA (B = −22.5, SE = 9.73 [95% CI: −41.7 to −3.32]), and PUFA (B = −14.0, SE = 5.39 [95% CI: −24.7 to −3.39]), while the intake of PUFA (B = 11.8, SE = 5.71 [95% CI: 0.60 to 23.1]) was associated positively with reading the nutrition fact labels of food products. For female students, reading the nutrition fact labels of food products was associated with a lower intake of SFA (B = −7.88, SE = 3.51 [95% CI: −14.8 to −0.94]). A simple linear regression analysis of the total score of behaviors related to limiting fat intake (independent variable) in relation to fat intake (dependent variable) was performed, and none of the models were statistically significant (see Table 4).

## 4. Discussion

The findings of our study indicated a significantly higher proportion of females reducing their use of fats and oils while cooking, as well as limiting their consumption of fatty foods (e.g., fried food items and fatty meats), compared to males. The median total score of behaviors related to limiting fat intake was significantly higher among females and supplement users, compared to other groups. Healthy weight, overweight, and obese students reported a significantly higher score of behaviors related to limiting fat intake compared to underweight students. Males who reported making an effort to limit their consumption of fatty foods consumed less total fat, SFA, MUFA, and PUFA, while those who reported reading the nutrition facts labels of food products consumed more PUFA. Females who reported reading the nutrition fact labels consumed less SFA.

Females tend to be more concerned with their body image and weight status than males, leading them to make more effort to limit fat intake [17]. In the current study, we found that females made more effort to limit fat intake compared to males. Specifically, females made more efforts to reduce their use of fats and oils while cooking and limit their intake of high-fat foods. In fact, males tended to consume more fatty foods (e.g., fast food). Recent research conducted among university students reported higher fast food consumption among male students, compared to female students [18]. It has been reported that females prefer to follow healthier diets, such as a vegetarian diet, whereas male students prefer to consume less healthy foods, including processed foods and alcohol [19]. Similarly, studies conducted among university students in Saudi Arabia reported a higher consumption of fast food among males compared to female students [20,21].

Behaviors related to limiting fat intake include, but are not limited to, consumption of reduced-fat dairy products, trimming visible fat and removing skin from meat and poultry, comparing food labels to choose foods that are lower in fat, and choosing leaner cuts of meat [22]. A previous cross-sectional study conducted in Saudi Arabia in 2021 found that females tended to use nutrition fact labels to inform their purchases more often than males [23]. However, in the current study, we found similar efforts to limit fat intake by reading nutrition labels among male and female students. A lack of interest in knowing the nutritional content of foods and unclear font and design have been cited as the most common reasons for not reading nutrition fact labels [24]. It has been suggested that individuals who read nutrition labels tend to be more concerned with the quality of purchased food products and their overall diet. A study conducted among female students in Saudi Arabia found that the majority of students who frequently read nutrition fact labels agree on the importance of the health value of food products [25]. Another study found that reading nutrition labels was associated with higher consumption of healthy foods and a lower percentage of total calories from SFA [26].

Several countries, such as the United States and Scotland, monitor fat intake at the population level [27,28]. A number of public health organizations emphasize the importance of adherence to the recommended daily intake levels of dietary fat. According to the recommendations of the Institute of Medicine, total fat intake should be ≤35% of total energy intake for adults; a further reduction to <30% of total energy intake is recommended [4,29]. Further, limiting the intakes of SFAs, trans-fatty acids, and dietary cholesterol has been suggested to improve the health status of individuals [4,29,30]. The WHO recommends the population to monitor and limit fat intake in order to prevent negative health consequences related to obesity and non-communicable diseases [31].

In this study, students within the healthy weight range and students with overweight and obesity reported a significantly higher score of behaviors related to limiting fat intake compared to underweight students. Perhaps, underweight students are not making efforts to limit fat intake in order to gain weight; however, intentions for weight reduction were not evaluated in this study. 

Excessive weight is a serious health-related issue that can influence individuals’ quality of life [32]; thus, efforts to limit fat intake can be a very simple approach to reduce weight. In addition, efforts to reduce fat intake might be the reason why individuals within the healthy weight range are in this weight category, despite the limited quality of diet followed by the general population [33].

Interventions have been designed to reduce fat intake in several settings, including Saudi Arabia. For example, the Saudi Food and Drug Authority have established food labeling-related policies that aimed to increase the consumer’s awareness concerning the nutrient content of food products and to improve food choices [12]. An intervention study that targeted pre-diabetic Saudi females included three groups: a face-to-face education program, a WhatsApp education program, and a control group with no education program. Results showed a significant reduction in the intake of fat and energy in the groups who received a face-to-face education program and a WhatsApp education program, while there was no significant reduction in fat and energy intake observed in individuals assigned to the control group [34]. A randomized controlled trial in New Zealand studied the effect of nutrition education and discounted pricing on purchasing healthy products from supermarkets, and the findings showed no effect of both interventions on the intake of total fat, SFA, and USFA [35]. A better understanding of the current behaviors related to fat intake can help in designing effective interventions that aim to limit the consumption of fat.

To the best of our knowledge, this is the first study in Saudi Arabia and the Middle East to investigate behaviors related to limiting fat intake, specifically among young adults who typically consume high amounts of fat. However, the present study is limited by the lack of data concerning energy intake, thus the percentage of fat consumed was not calculated. Another limitation is the cross-sectional design used in our study, which did not allow us to infer causality between fat-limiting behaviors and actual fat intake. Lastly, the generalizability of the findings are limited due to the collection of data from a single university. Future work should be directed towards providing data concerning fat intake and behaviors related to limiting fat intake among the general population of Saudi Arabia and across the different age groups and socioeconomic status.

## 5. Conclusions

Efforts to limit fat intake have been noted, especially among females; however, these efforts were not linked to fat intake among young adults in Saudi Arabia. Interventions that aim to increase awareness concerning fat food sources and how to read nutrition fact labels are needed. Future longitudinal studies should be conducted to monitor the fat intake among the Saudi population and to investigate environmental factors associated with high fat intake, to prevent health issues related to the high consumption of fat.

## Figures and Tables

**Table 1:** Sample characteristics (*n* = 305).

Variable	*n*	%
Sex		
Male	152	49.8
Female	153	50.2
Age group		
19–20 years	141	46.2
21–23 years	146	47.9
24–26 years	18	5.90
Marital status		
Single	292	95.7
Married	13	4.30
Family monthly income		
<SR 6000	95	31.1
SR 6000–10,999	76	24.9
SR 11,000–15,999	56	18.4
SR 16,000–20,999	43	14.1
≥SR 21,000	35	11.5
Employment status		
Employed	21	6.90
Unemployed	284	93.1
Living status		
With family	290	95.1
Alone	12	3.90
Dormitory	3	1.00
Year of study		
1st or 2nd year	91	29.8
3rd or 4th year	179	58.7
5th to 7th year	35	11.5
Major		
Health-related field	139	45.6
Non-health-related field	166	54.4
Sleeping hours per day		
<7 h	112	36.7
≥7 h	193	63.3
Media use per day		
≤2 h	17	5.60
>2 h	288	94.4
Supplement use		
Yes	61	20.0
No	244	80.0
Weight status		
Underweight	57	18.7
Healthy weight	157	51.5
Overweight	59	19.3
Obese	32	10.5

**Table 2:** Behaviors related to limiting fat intake (*n* = 305).

Variable	Male Students (*n* = 152)	Female Students (*n* = 153)	*p*-Value
Read nutrition fact labels			
No	119 (50.0)	119 (50.0)	1.000
Yes	33 (49.3)	34 (50.7)	
Consume low-fat milk and dairy products			
No	134 (51.0)	129 (49.0)	0.419
Yes	18 (42.9)	24 (57.1)	
Reduce the use of fats and oils while cooking			
No	115 (55.8)	91 (44.2)	<0.001 *
Yes	37 (37.4)	62 (62.6)	
Limit the consumption of fast food			
No	87 (55.1)	71 (44.9)	0.075
Yes	65 (44.2)	82 (55.8)	
Limit the consumption of fatty food (e.g., fried food items and fatty meats)			
No	114 (59.7)	77 (40.3)	<0.001 *
Yes	38 (33.3)	76 (66.7)	

* Statistically significant with alpha = 0.05. *p*-values presented in this table were obtained from Fisher’s exact test.

**Table 3:** Association between characteristics of the study sample and behaviors related to limiting fat intake (*n* = 305).

Variable	Mean ± SD	Median (IQR)
Sex		
Male	1.26 ± 1.24	1.00 (0.00–2.00)
Female	1.82 ± 1.32	2.00 (1.00–3.00)
*p*-value	<0.001 *	
Age group		
19–20 years	1.50 ±1.24	1.00 (0.00–2.00)
21–23 years	1.58 ± 1.36	1.00 (0.00–3.00)
24–26 years	1.44 ± 1.38	1.00 (0.00–2.25)
*p*-value	0.888	
Marital status		
Single	1.53 ± 1.30	1.00 (0.00–2.00)
Married	1.77 ± 1.48	1.00 (0.50–3.00)
*p*-value	0.559	
Family monthly income		
<SR 6000	1.52 ± 1.33	1.00 (0.00–3.00)
SR 6000–10,999	1.54 ± 1.18	1.50 (1.00–2.00)
SR 11,000–15,999	1.38 ± 1.27	1.00 (0.00–2.00)
SR 16,000–20,999	1.53 ± 1.47	1.00 (0.00–3.00)
≥SR 21,000	1.86 ± 1.35	2.00 (1.00–3.00)
*p*-value	0.536	
Employment status		
Employed	1.48 ± 1.12	1.00 (0.50–2.50)
Unemployed	1.54 ± 1.32	1.00 (0.00–2.75)
*p*-value	0.989	
Living status		
With family	1.54 ± 1.28	1.00 (0.00–2.25)
Alone	1.41 ± 1.62	1.00 (0.00–2.75)
Dormitory	1.66 ± 2.08	1.00 (0.00–na)
*p*-value	0.841	
Major		
Health-related field	1.58 ± 1.34	1.00 (0.00–2.00)
Non-health-related field	1.51 ± 1.28	1.00 (0.00–3.00)
*p*-value	0.772	
Sleeping hours per day		
<7 h	1.61 ± 1.43	1.00 (0.00–3.00)
≥7 h	1.50 ± 1.23	1.00 (0.00–2.00)
*p*-value	0.721	
Media use per day		
≤2 h	1.76 ± 1.52	2.00 (0.00–3.00)
>2 h	1.52 ± 1.29	1.00 (0.00–2.00)
*p*-value	0.542	
Supplement use		
Yes	2.00 ± 1.33	2.00 (1.00–3.00)
No	1.42 ± 1.28	1.00 (0.00–2.00)
*p*-value	0.002 *	
Weight status		
Underweight	1.00 ± 1.27	0.00 (0.00–2.00)
Healthy weight	1.63 ± 1.27	2.00 (1.00–3.00)
Overweight	1.71 ± 1.30	2.00 (1.00–3.00)
Obese	1.72 ± 1.37	2.00 (1.00–2.75)
*p*-value	0.003 *	

* Statistically significant with alpha = 0.05. *p*-values presented in this table were obtained using Mann–Whitney and Kruskal–Wallis tests.

**Table 4:** Simple linear regression analysis of association between behaviors related to limiting fat and fat intake among male and female students.

Variable	Beta	Standard Error	95% Confidence Interval	*p*-Value	R-Square	Beta	Standard Error	95% Confidence Interval	*p*-Value	R-Square
Male Students						Female Students				
Total fat										
Read nutrition fact labels	54.8	29.6	−3.75 to 113	0.066	0.02	−12.8	10.0	−32.7 to 7.10	0.206	0.01
Consume low-fat milk and dairy products	−32.1	38.2	−107 to 43.3	0.402	0.01	21.2	11.4	−1.39 to 43.8	0.066	0.02
Reduce the use of fats and oils while cooking	−29.7	28.7	−86.4 to 26.9	0.302	0.08	−6.45	8.56	−23.4 to 10.4	0.452	0.00
Limit the consumption of fast-food	−35.3	24.8	−84.3 to 13.7	0.157	0.01	−1.31	8.44	−18.0 to 15.3	0.876	0.00
Limit the consumption of fatty food	−68.9	27.9	−124 to −13.6	0.015 *	0.04	−3.71	8.41	−20.4 to 12.9	0.659	0.00
Saturated fatty acid										
Read nutrition fact labels	13.8	9.86	−5.68 to 33.3	0.163	0.01	−7.88	3.51	−14.8 to –0.94	0.026 *	0.03
Consume low-fat milk and dairy products	−8.01	12.7	−33.0 to 17.0	0.528	0.00	6.15	4.04	−1.84 to 14.1	0.130	0.02
Reduce the use of fats and oils while cooking	−11.8	9.49	−30.6 to 6.93	0.215	0.01	−3.35	3.01	−9.29 to 2.59	0.267	0.01
Limit the consumption of fast-food	−10.5	8.23	−26.8 to 5.71	0.202	0.01	−1.80	2.97	−7.67 to 4.06	0.545	0.00
Limit the consumption of fatty food	−23.3	9.26	−41.6 to −5.02	0.013 *	0.04	−3.81	2.95	−9.64 to 2.01	0.198	0.01
Monounsaturated fatty acid										
Read nutrition fact labels	19.6	10.2	−0.64 to 39.9	0.058	0.02	−2.13	3.68	−9.41 to 5.14	0.564	0.00
Consume low-fat milk and dairy products	−13.1	13.2	−39.2 to 13.1	0.325	0.01	6.22	4.18	−2.04 to 14.4	0.139	0.01
Reduce the use of fats and oils while cooking	−8.19	9.96	−27.8 to 11.5	0.413	0.00	−1.41	3.12	−7.58 to 4.75	0.650	0.00
Limit the consumption of fast-food	−12.6	8.60	−29.6 to 4.37	0.144	0.01	1.41	3.07	−4.65 to 7.48	0.646	0.00
Limit the consumption of fatty food	−22.5	9.73	−41.7 to –3.32	0.022 *	0.04	1.40	3.06	−4.65 to 7.46	0.648	0.00
Polyunsaturated fatty acid										
Read nutrition fact labels	11.8	5.71	0.60 to 23.1	0.039 *	0.03	−1.40	2.26	−5.87 to 3.07	0.537	0.00
Consume low-fat milk and dairy products	−9.32	7.35	−23.8 to 5.20	0.207	0.01	5.10	2.56	0.04 to 10.16	0.048	0.03
Reduce the use of fats and oils while cooking	−3.09	5.56	−14.0 to 7.89	0.579	0.00	−0.83	1.92	−4.631 to 2.957	0.663	0.00
Limit the consumption of fast-food	−8.00	4.78	−17.4 to 1.44	0.096	0.02	−0.21	1.89	−3.952 to 3.522	0.910	0.00
Limit the consumption of fatty food	−14.0	5.39	−24.7 to –3.39	0.010 *	0.04	−0.06	1.88	−3.79 to 3.665	0.974	0.00
Total behavior related practices to limit fat intake										
Total fat	−13.9	9.96	−33.5 to 5.82	0.166	0.01	−1.29	3.21	−7.64 to 5.05	0.688	0.00
Saturated fatty acid	−5.02	3.30	−11.5 to 1.50	0.130	0.02	−1.61	1.12	−3.83 to 0.62	0.155	0.01
Monosaturated fatty acid	−4.51	3.46	−11.3 to 2.33	0.194	0.01	0.48	1.70	−1.84 to 2.79	0.685	0.00
Polyunsaturated fatty acid	−2.71	1.93	−6.51 to 1.10	0.162	0.01	0.09	0.72	−1.33 to 1.52	0.896	0.00

* Statistically significant with alpha = 0.05.

## Data Availability

The datasets used and/or analyzed during the current study are available from the corresponding author on reasonable request.

## Appendix A

**Table A1:** Fat intake of male and female undergraduate students (*n* = 305).

Variables	Male Students (*n* = 152)		Female Students (*n* = 153)		*p*-Value
	Mean ± SD	Median (IQR)	Mean ± SD	Median (IQR)	
Total fat, g	149 ± 152	95.2 (59.0–169)	90.1 ± 51.9	78.9 (53.4–112)	0.001 *
Saturated fatty acid, g	51.3 ± 50.3	32.9 (21.7–62.2)	32.3 ± 18.2	29.9 (18.4–39.7)	0.005 *
Monounsaturated fatty acid, g	49.1 ± 52.6	29.9 (18.7–53.3)	30.8 ± 18.9	26.1 (17.4–38.6)	0.022
Polyunsaturated fatty acid, g	28.1 ± 29.3	16.7 (10.6–33.7)	16.0 ± 11.6	12.7 (8.51–19.2)	<0.001 *
Fat from plant food sources, g	70.4 ± 84.0	39.0 (22.8–88.5)	43.4 ± 31.9	34.9 (20.1–52.1)	0.051
Fat from animal food sources, g	81.8 ± 87.1	49.8 (32.1–94.5)	46.7 ± 28.7	39.2 (27.6–58.5)	<0.001 *

* Statistically significant with alpha = 0.008. *p*-values presented in this table were obtained from Mann–Whitney test.
